# Fumarate induces LncRNA-MIR4435-2HG to regulate glutamine metabolism remodeling and promote the development of FH-deficient renal cell carcinoma

**DOI:** 10.1038/s41419-024-06510-2

**Published:** 2024-02-19

**Authors:** Liangsong Zhu, Yilun Hong, Ziran Zhu, Jiwei Huang, Jianfeng Wang, Ge Li, Xiaoyu Wu, Yonghui Chen, Yunze Xu, Liang Zheng, Yiran Huang, Wen Kong, Wei Xue, Jin Zhang

**Affiliations:** 1 Department of Urology, Ren Ji Hospital, School of Medicine, Shanghai Jiaotong University, Shanghai, China; 2 Department of Pediatric Translational Medicine Institute, Shanghai Children’s Medical Center, School of Medicine, Shanghai Jiao Tong University, Shanghai, China

**Keywords:** Cancer, Cell biology

## Abstract

Fumarate hydratase (FH) deficient renal cell carcinoma (RCC) is a type of tumor with definite metabolic disorder, but the mechanism of metabolic remodeling is still unclear. LncRNA was reported to closely correlate with cancer metabolism, however the biological role of LncRNA in the development of progression of FH-deficent RCC was not well studied either. FH-deficient RCC samples were collected in my hospital and used for RNA-sequencing and Mass spectrometry analysis. FH-deficient RCC cell line UOK262 and control pFH cells were used for in vitro experiments, including proliferation assay, transwell assay, western-blot, mass spectrometry and so on. PDX mouse model was used for further drug inhibition experiments in vivo. In this study, we analyzed the profiles of LncRNA and mRNA in FH-deficienct RCC samples, and we found that the LncRNA-MIR4435-2GH was specifically highly expressed in FH-deficient RCC compared with ccRCC. In vitro experiments demonstrated that MIR4435-2HG was regulated by Fumarate through histone demethylation, and the deletion of this gene could inhibit glutamine metabolism. RNA-pulldown experiments showed that MIR4435-2HG specifically binds to STAT1, which can transcriptionally activate GLS1. GLS1 inhibitor CB-839 could significantly suppress tumor growth in PDX tumor models. This study analyzed the molecular mechanism of MIR4435-2HG in regulating metabolic remodeling of FH-deficient RCC in clinical samples, cells and animal models by combining transcriptional and metabolic methods. We found that that GLS1 was a therapeutic target for this tumor, and MIR4435-2HG can be used as a drug sensitivity marker.

## Background

Abnormal cell proliferation is the basic feature of tumor development, and abnormal tumor metabolism is also the basic manifestation of this feature [1, 2]. It is well known that in the occurrence and development of renal cell carcinoma (RCC), there are dozens of genes involved in the reactions related to RCC metabolism, including the awareness of oxygen in the microenvironment, the uptake of iron and the uptake and utilization of nutrients, among which the most important one related to glucose metabolism is Tricarboxylic Regulation of fumarate hydrase (FH) and Succinate dehydrogenase (SDH) in TCA cycle [3]. Hereditary leiomyomatosis and renal cell carcinoma (HLRCC) is a kind of more aggressive RCC with germline loss-of-function mutation of *FH* [4]. HLRCC is a kind of autosomal dominant genetic disease syndrome, patients usually have uterine fibroids disease and RCC, the kidney pathological morphological characteristics similar to the type II papillary renal cell carcinoma (pRCC), and the tumor becomes more aggressive and higher malignant degree, metastasis may occur when tumor is small [5, 6]. This kind of RCC was also named as the FH-deficient RCC. The basic cause of this disease is that the loss-function-mutation of FH gene leads to the failure of the normal encoding of Fumarate hydratase, thus causing the accumulation of fumarate in the cell microenvironment. Fumarate was proved to be a key role in cell transformation and tumourgenesis [7]. It has been reported that fumarate can also act as an epigenetic regulator. On the one hand, fumarate can regulate the demethylation of DNA and histones by inhibiting the a-ketoglutarate-dependent dioxygenase activity [8–10]. On the other hand, fumurate can promote tumor metastasis by inhibiting the demethylation of miRNA functional regions, thereby up-regulating the expression of EMT-related transcription factors negatively regulated by miRNA [11, 12]. Long non-coding RNA (LncRNA) is a class of RNA with a length greater than 200nt and does not have the ability to encode protein, LncRNA also belongs to the category of epigenetic inheritance [13]. In recent years, with the development of metabonomics, it has been found that the Crosstalk between LncRNA and tumor cell metabolism is closely related to the progress and metastasis of cancer, and it is very important to explore the internal relationship between them [14]. However, the metabolic regulation of LncRNA in FH-deficient renal carcinoma and the specific mechanism of promoting its malignant progression are still unclear and need further research.

In order to find out the specific oncogenic LncRNAs in FH-deficient RCC, we examined genome-wide LncRNA and mRNA expression in 3 paired of FH-deficient RCC samples and paired-normal tissues. In addition, tumor and paracancer specimens from 4 clear cell RCC (ccRCC) patients were used as controls. And we identified that the LncRNA-MIR4435-2HG was specifically highly expressed in FH-deficient RCC instead of ccRCC. We also demonstrated that higher MIR4435-2HG expression was significant correlated with advanced tumor stage and poor survival in pRCC patients. Moreover, the in vitro and in vivo experiments showed that MIR4435-2HG plays a key role in glutamine metabolism remodeling and cancer development by promoting STAT1/GLS1 signal pathway, suggesting that targeting GLS1 by specific inhibitor CB-839 may be a promising therapeutic strategy for FH-deficient RCC treatment.

## Materials and methods

### Patients and tissues

Eight HLRCC patients and six ccRCC patients who take radical nephrectomy in our hospital were collected and enrolled in this study, the pathological characteristics were confirmed by pathological section and immunohistochemistry analysis (Supplementary Table S1). Tissues from three of the HLRCC patients and four of the ccRCC patients were used for RNA-sequencing. The rest fresh samples from these patients were divided into several parts, one for qPCR experiments, one for mass spectrometry to detect metabolites and other for patient-derived xenograft models. Written informed consent was obtained from each participant.

### Bioinformatics analysis

Three paired HLRCC samples and four paired ccRCC samples were used for RNA-sequencing analysis at Majorbio Company (Shanghai, China). The gene sets showing FDR, 0.25, a well-established cutoff for the identification of important genes, were considered enriched between the classes under comparison. And the online website of lncRNA cloud analysis (www.majorjob.com) was used for the enrichment analysis.

### FISH assay

Fluorescence in situ hybridization (FISH) experiments were performed to detect the LncRNA expression in tissue sections by standard protocol, and the specific MIR4435-2HG FISH probes conjugated with Digoxin as follows in list it was designed and synthesized by Exonbio (Gungzhou, China). Then anti-Dig with HRP and TSA to development the signal (Supplementary Table S2).

### Cell lines and cell culture

*Fh1*-deficient cells (uok262) and *Fh1*-proficient cells (pFH) were kindly provided by Prof. Liang Zheng [15]. All cell lines were cultured using DMEM (Hyclone, Cat# SH30081) supplemented with 10% heat-inactivated serum (Gibco, Cat# 16170078) and 2 mM glutamine (Gibco, Cat# 25030149) and 1 mM pyruvate (Gibco, Cat# 11360070) and were regularly tested to ensure they were mycoplasma free using a mycoplasma detection kit (R&D, Cat# CUL001B). The other renal tumor cell lines such as 786-O, 769-p, ACHN and CAKI-1, and the normal renal tubular epithelial cell line HK2 were purchased from American Type Culture Collection (ATCC, Manassas, VA, USA). ACHN cell line was cultured in MEM media with 10% heat-inactivated serum, and other cell lines were cultured with 1640 media contained 10% heat-inactivated serum. All cell lines were maintained at 37 °C with 5% CO_2_.

### RNA extraction and quantitative RT-PCR

The total RNAs of HLRCC, ccRCC samples and cell lines were isolated by using TRIZOL reagent (Invitrogen) according to the standard protocol. And the quantification of LncRNA and histone methyl transferase were performed with the SYBR Green kit (Takara Bio, Dalian, China). The primer sequences were listed in supplementary Table S3.

### Cell lines with siRNA interference and lentiviral transfection

The siRNA transfection screen was used to detect the most valuable LncRNA in HLRCC. The siRNA and negative control were transfected with lipofectamine RNAiMAX reagent (Invitrogen, Carlsbad, CA, USA) following the standard manufacturer’s instructions.

The lentivirus expression human MIR4435-2HG (LV-MIR4435-2HG) and control lentivirus were designed and purified by GeneChem Group (shanghai, China). New stable HLRCC cell lines were established after puromycin cultivating and used in further research. All the sequences were listed in supplementary Table S4.

### Cell proliferation and inhibitor experiments

Cells were plated into 6-well plates and incubated overnight at 37 °C (siRNA transfection was performed after the cells were fully adherent), then cell counts by using electronic cell counter were taken every 24 h for 96 h post incubation.

GLS1 inhibitor CB-839 (HY-12248) and LWG-301 (HY-152207) were purchased from MedChemExpress. The inhibition of CB839 and LWG-301 on HLRCC cell lines were determined by sulforhodamine B (SRB) assay according to the standard protocol as described previously [16]. All procedures were repeated three times.

### Cell colony formation assay

Cultured HLRCC cell lines were seed in 6-well plates with a density of 1000 cells per well. Cell colony formation was measured after 7 days and stained with crystal violet, photographed and counted when the wells were fully dried.

### Transwell migration assay

Cells were seeded in the 8.0 μm size transwell inserts (Corning, Lowell, MA, USA) with appropriate number respectively. The upper migration stoppers were cultured with serum-free DMEM medium while the bottom wells were cultured with 10%-serum medium. The invaded cells of each cell lines were fixed with 95% methanol and stained with crystal violet according to the standard protocol.

### Metabolic mass spectrometry assay

Cells at the treatment end point (500,000 to 1,000,000 cells per sample) were collected and added with 80% ice methanol, then the supernatant was taken by centrifugation at 12,000 after shaking at 4 °C for 30 min for mass spectrometry analysis. Metabolomics analysis was performed by ultra-high performance liquid chromatography tandem mass spectrometry (LC-MS), in which metabolites were separated by Ultimate 3000 liquid chromatography ZIC-pHILIC column, and metabolites were detected by full scan with positive and negative switching scanning modes after final sample collection.

### RNA-pulldown assay

In this experiment, we used the F2-RNA pull-down test kit (FI88702, FITGENE company, Guangzhou, China). Firstly, we constructed the F2 tagged MIR4435-2HG (Supplementary Table S5), and transfected into host cells. Magnetic beads preparation and RNA-binding protein extraction were performed according to the standard kit instructions. Then the extracted protein complex was used for subsequent SDS-PAGE and western-blot experiments.

### Chromatin isolation by RNA purification

Chromatin isolation by RNA purification (ChIRP) was performed by using ChIRP kit (BersinBio) according to the standard protocol. The chromatin was cross-linked and fragmented by sonication, then the chromatin containing the MIR4435-2HG with biotin-labeled probe was pulled down by using streptavidin-conjugated magnetic beads. Western-blot was using to analyze the immunoprecipitated chromatin.

### Western-blot assay

Western-blot experiment was performed according to the standard protocol. The membranes were incubated with primary antibodies: H3K4me1(Cat.5326 T), H3K4me2(Cat.4658 T), H3K4me3(Cat.4909 T), H3K27ac(Cat.8173 T), STAT1(Cat.14994 T), GLS1(Cat.56750 T), PARP(Cat.9532 T),β-actin (Cat.4967 S) (CST, Boston, MA, USA) overnight at 4 °C. Then the membranes were washed and incubated with secondary antibody at room temperature for 2 h. The target protein bands were detected by using ECL agents with enhanced chemiluminescence method in ChemiScope3400 imaging system.

### Chromatin immunoprecipitation assay

Chromatin immunoprecipitation was performed according to the manufacture’s protocol (SimpleChIP Plus Enzymatic Chromatin IP Kit9005, CST, Boston, MA, USA). The relative H3K4me3(Cat.4909 T) enrichment of MIR4435-2HG promoter was normalized against that in the input samples, while the binding site enrichment of transcript factor STAT1 and GLS1 was compared with control group and si-STAT1 group.

### Cut and Tag experiment

Cut&Tag sequencing was commissioned by the NeoBio company (shanghai) according to the standard procedure [17]. Cells collecting and DNA library construction were performed according to the manufacture’s protocol. Then the sequencing was performed by Illumina Nova6000. A more detailed step-by-step protocol can be found at https://www.protocols.io/view/bench-top-cut-amp-tag-wnufdew/abstract.

### Patient-derived xenograft model and treatments

Immunodeficient NOD-SCID gamma mice (NSG) were used for patient derived xenograft experiments. Fresh tumor tissues from HLRCC patients were collected and mixed with sterile PBS that contained 1% (vol/vol) pen-strep solution. Then the tissues were cut to minimum fragments (~1.5 mm) for subcutaneous implantation. Tumor growth was monitored and measured twice a week and GLS1 inhibitor (CB-839) 100 mg/kg or normal saline was intragastric administration when the tumor reached 200 mm^3^. Animals were killed after 3-weeks drug treatments, and the tumor weight was measured in the end of in vivo experiments. The tumors were saved in 4% paraformaldehyde and used for IHC staining analysis, such as anti-FH(Proteintech, Cat.11375-1-AP), anti-2SC(Discoveryantibodies, Cat.crb2005017) and anti-Ki67(CST, Cat.9027 T). All animal experiments procedures were approved by the Animal Care and Use Committee of Renji Hospital, Shanghai Jiaotong University (Shanghai, China).

### Statistic analysis

All statistical analyses were performed with GraphPad Software version 7.0 (San Diego, CA, USA). Significant difference between different groups were tested by using a two-tailed, paired t-test analysis. All p values of less than 0.05 were considered significance.

## Results

### Identification of MIR4435-2HG as an oncogenic lncRNA in FH-deficient RCC

**Fig. 1:**
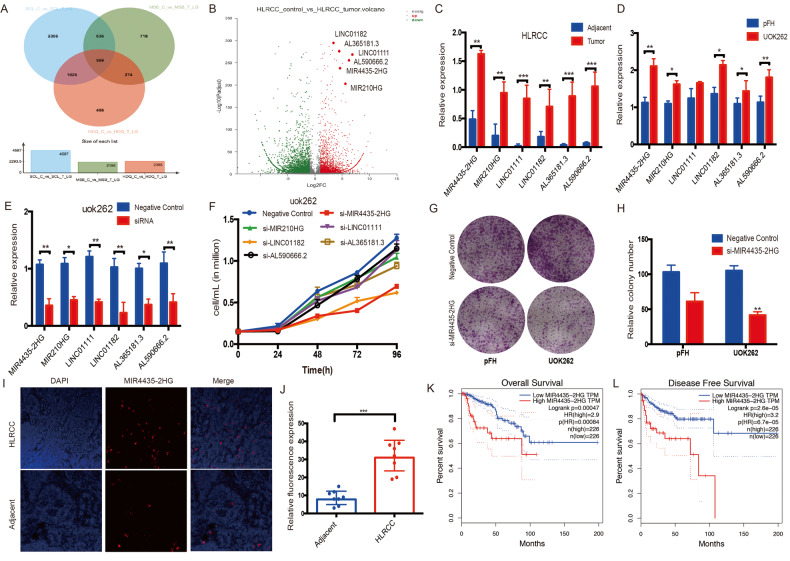
LncRNA-MIR4435-2HG is determined to be an oncogenic factor in FH-deficient RCC. **A**, **B** The bioinformation analysis showed upregulated lncRNAs in FH-deficient RCC tissues. **C**, **D** LncRNAs’ expression determined both in fresh tumor samples and cell lines. **E** siRNA screeing was performed to select the candidate lncRNA. **F** Proliferation rate was analyzed after siRNA transfection. **G**, **H** Comparing clone formation ability between MIR4435-2HG silence and control group. **I, J** FISH assay was performed to determine to expression of MIR4435-2HG in fresh samples. **K**, **L** Survival was analyzed and compared between patients with high and low level of MIR4435-2HG in pRCC patients according to the public database. *p < 0.05, **p < 0.01, ***p < 0.001.

Three paired HLRCC samples and four ccRCC samples were used to find out the oncogenic lncRNAs by high-throughput sequencing (Supplement Fig. S1A, B). According to the lncRNA and mRNA expression profile, we sorted 596 lncRNAs by intersection of three HLRCC patients (FDR < 0.005, Fold change > 3) (Fig. 1A, B). Next, we selected the top lncRNAs for further cell line experiments, and the sequencing results were verified by qPCR by fresh samples and cell lines (Fig. 1C, D). The results showed that Lnc-MIR4435-2HG, Lnc-MIR210HG, LINC01111, LINC01182, Lnc-AL365181.3 and Lnc-AL590666.2 were highly expressed both in HLRCC samples and FH-deficient RCC cell lines. The lncRNA subcellular location was predicted by online website (LncLocator: *lncRNA subcellular localization predictor*) (Supplement Fig. S1C). Then we designed and used siRNA knocking down system to determine the key lncRNAs for proliferation of FH-deficient RCC (Fig. 1E, Supplementary Table S1). And we found that MIR4435-2HG knocking down significantly decreased cell growth both in CCK8 assay and cell clone experiment, which indicate that this gene plays an important role in the development of FH-deficient RCC (Fig. 1F, G). Further, we searched the GEPIA database and found MIR4435-2HG was negatively correlated with FH expression (Supplementary Fig. S1E). We also tested the MIR4435-2HG by FISH assay in HLRCC samples, the results showed MIR4435-2HG was significantly overexpressed in HLRCC cancer tissues compared with adjacent tissues (Fig. 1I, J). This result was verified by KIRP database, and MIRR4435-2HG was significantly correlated with advanced tumor stage of pRCC (Supplementary Fig. S1F, G). OS and DFS survival analysis also showed that high MIRR4435-2HG expression correlated with worse prognosis (Fig. 1K, L). In summary, MIRR4435-2HG is specifically overexpressed in FH-deficient RCC cancer tissues and is closely related to the clinical prognosis.

### Fumarate induces MIR4435-2HG expression by H3K4me3 modification

**Fig. 2:**
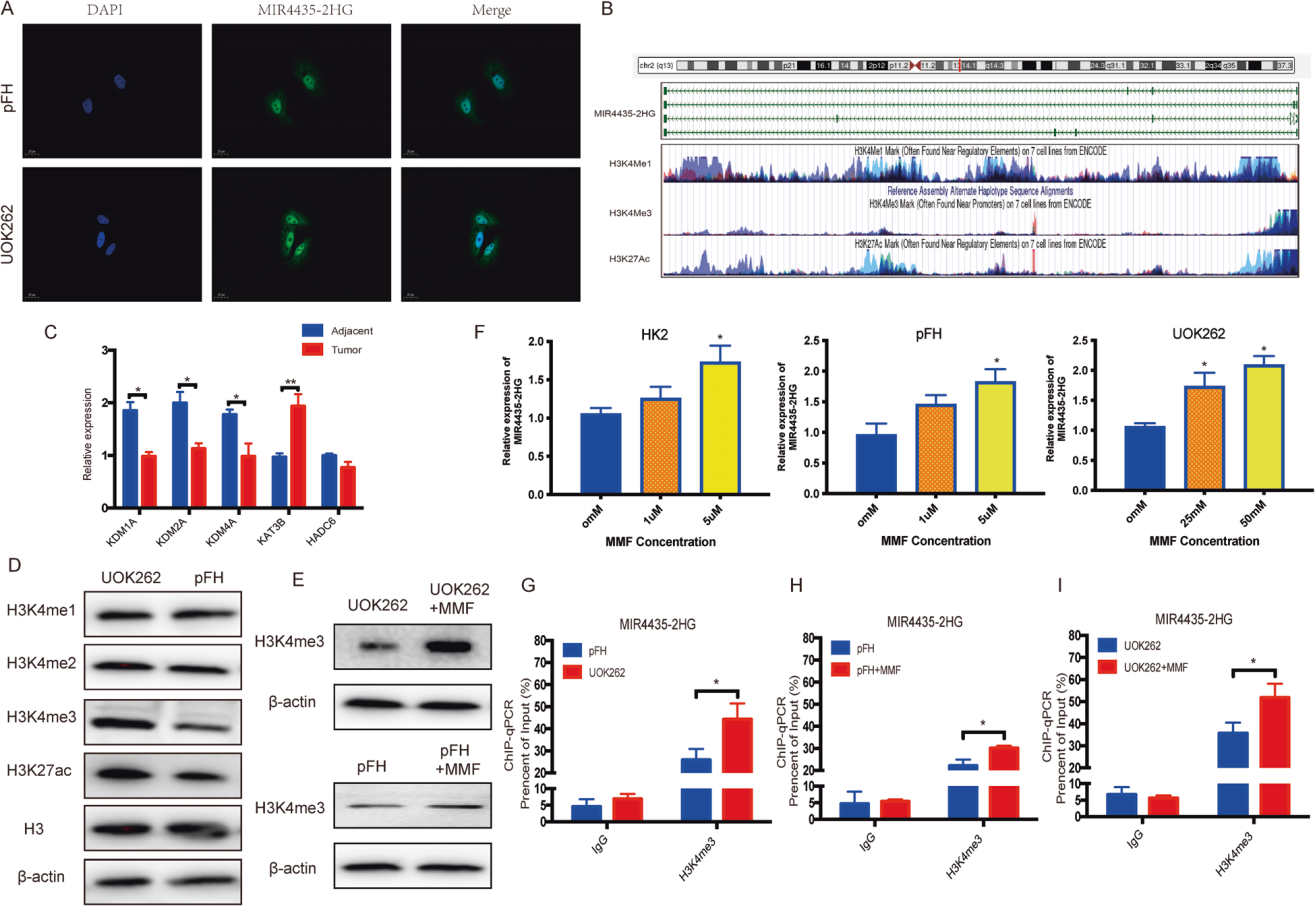
Fumarate induced MIR4435-2HG expression by H3K4me3 modification. **A** The representative images of cellular immunofluorescence to detect the MIR4435-2HG localization in FH-deficient cancer cells. **B** The specific enrichment of H3K4 methylation and H3K27 acetylation peaks in the promoter region of MIR4435-2HG according to the UCSC Genome Bioinformatics Site (http://genome.ucsc.edu/). **C** The expression of common demethylases and deacetylase in HLRCC tissues. **B** The H3K4me1, H3K4me3, H3K27Ac enrichment in MIR4435-2HG. **D** The western-blot analysis was performed to determine the H3 methylation and acetylation level in cell lines. **E** H3K4me3 level was analyzed after MMF treatment. **F** qPCR was performed in HK2, pFH and UOK262 cells treated with MMF. **G**–**I** ChIP assay was performed to determine the H3K4me3 level in MIR4435-2HG promoter region. *p < 0.05, **p < 0.01, ***p < 0.001.

We firstly used cellular immunofluorescence to detect the MIR4435-2HG localization and found that MIR4435-2HG was mainly localized in nucleus in FH deficient cancer cells (Fig. 2A). Then we explored the mechanisms underlying the high expression of MIR4435-2HG in FH-deficient RCC. In order to study whether fumarate modulated MIR4435-2HG up-expression by histone methylation, we searched UCSC Genome Bioinformatics Site (http://genome.ucsc.edu/), and found that the high enrichment of H3K4 methylation and H3K27 acetylation peaks in the promoter region of MIR4435-2HG (Fig. 2B). These results were showing a potential relationship between histone modification and MIR4435-2HG expression. We deeply investigated the transcriptome data, according to the RNA-sequence results and transcriptome analysis, we found that the demethylases such as KDM1A, KDM2A, KDM4A and HADC6 were significantly decreased in HLRCC samples (Fig. 2C). Then, we tested the H3K1me1, H3K4me2, H3K4me3 and H3K27ac expression in UOK262 and pFH cells. The western blot assay showed that H3K4me3 was significantly increased in UOK262 compared with pFH (Fig. 2D). Incubating cells with monomethyl fumarate (MMF), a kind of cell permeable derivative fumarate triggered profound FH-deficient cells phenotype, and found that MIR4435-2HG was increased as well as H3K4me3 expression (Fig. 2E, F). At the same time, we tested the expression of several H3K4 methyl transferases, found MLL1/KMT2A and MLL4/KMT2D were increased after MMF treatment (Supplementary Fig. S2A). ChIP assay was performed to determine the specific H3K4me3 modification on the promoter region of MIR4435-2HG. And the results showed that H3K4me3 was highly enriched in MIR4435-2HG promoter and MMF incubating would increase H3K4me3 level as well (Fig. 2G–I). Taken together, we thought that fumarate induced MIR4435-2HG expression by regulating H3K4me3 in FH-deficient RCC.

### MIR4435-2HG promotes FH-deficient RCC cell proliferation depends on glutamine reprograming

**Fig. 3:**
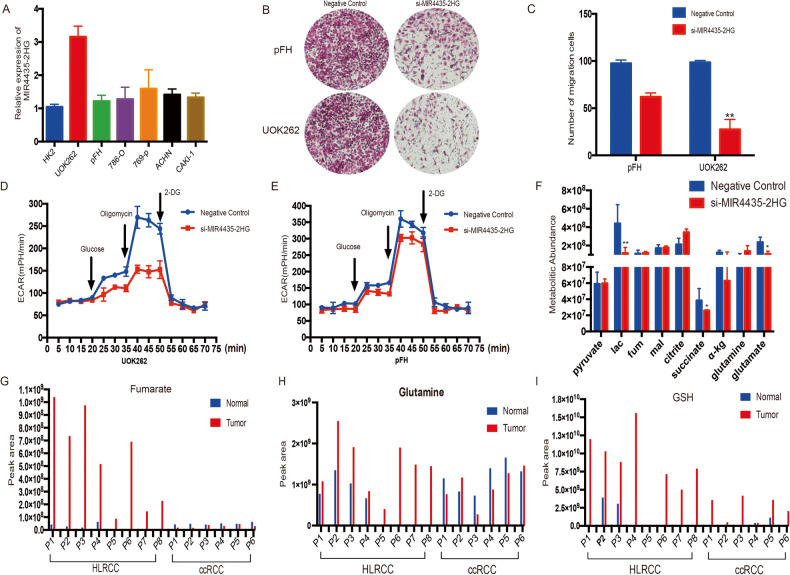
MIR4435-2HG inhibition decreased glycolysis and glutamine metabolism in FH-deficient RCC. **A** The expression level of MIR4435-2HG in different RCC cell lines. **B**, **C** Transwell assay was performed in pFH and UOK262 with MIR4435-2HG knocking down. **D**, **E** Extracellular acid ratio (ECAR) upon cells were measured after MIR4435-2HG knocking down in UOK262 and pFH cells. **F** Mass spectrometry detection was performed after MIR4435-2HG transfected with siRNA in UOK262 cells. **G**–**I** Mass spectrometry detection was performed in HLRCC and ccRCC samples. *p < 0.05, **p < 0.01, ***p < 0.001.

Firstly, we tested the MIR4435-2HG expression in FH-dificient RCC cell lines and other types of RCC cell lines, such as HK2, 786-O, 769-P, ACHN and CAKI-1. And found MIR4435-2HG was specifically overexpressed in UOK262 cells (Fig. 3A). Indeed, knockdown of MIR4435-2HG significantly suppressed cell migration in UOK262 (Fig. 3B, C). We next tested if knocking down MIR4435-2HG directly influenced the glycolytic metabolism and the changes of metabolites in UOK262 cells. The extracellular acidification rate (ECAR) measurement assay showed that MIR4435-2HG silence of MIR4435-2HG significantly reduced ECAR level in UOK262 compared with pFH cells (Fig. 3D, E). At the same time, metabolic mass spectrometry (MS) showed that lactate, a-KG and glutamate were obviously reduced after MIR4435-2HG knocking down in UOK262 cells (Fig. 3F). To further demonstrate the biological function of MIRR4435-2HG, GFP-labeled-lentivirus was used to increase MIR4435-2HG in FH-deficient RCC cells. In gain-of function assays, we found overexpression MIR4435-2HG could significantly promote cell proliferation and migration in UOK262 cells (Supplementary Fig. S2B–F). MS also showed that a-KG and glutamate were significantly increased in cells with LV-MIR4435-2HG transfection (Supplementary Fig. S2G). These data indicated that MIRR4435-2HG participated in promoting cancer development and regulating glutamine metabolism remodeling in FH-deficient RCC. Meanwhile, we performed MS experiments to test the metabolic levels in several tumor tissues (including 8 pairs HLRCC and 6 pairs ccRCC samples), as showed in Fig. 3G–I and Supplementary Fig. S3H–J, it was found that the expression levels of fumarate, glutamine and GSH were significantly up-regulated in FH-deficient RCC compared with ccRCC. Indicating that glutamine metabolism was more active in FH-deficient RCC.

### MIR4435-2HG interacted with STAT1 and transcriptional activated GLS1

**Fig. 4:**
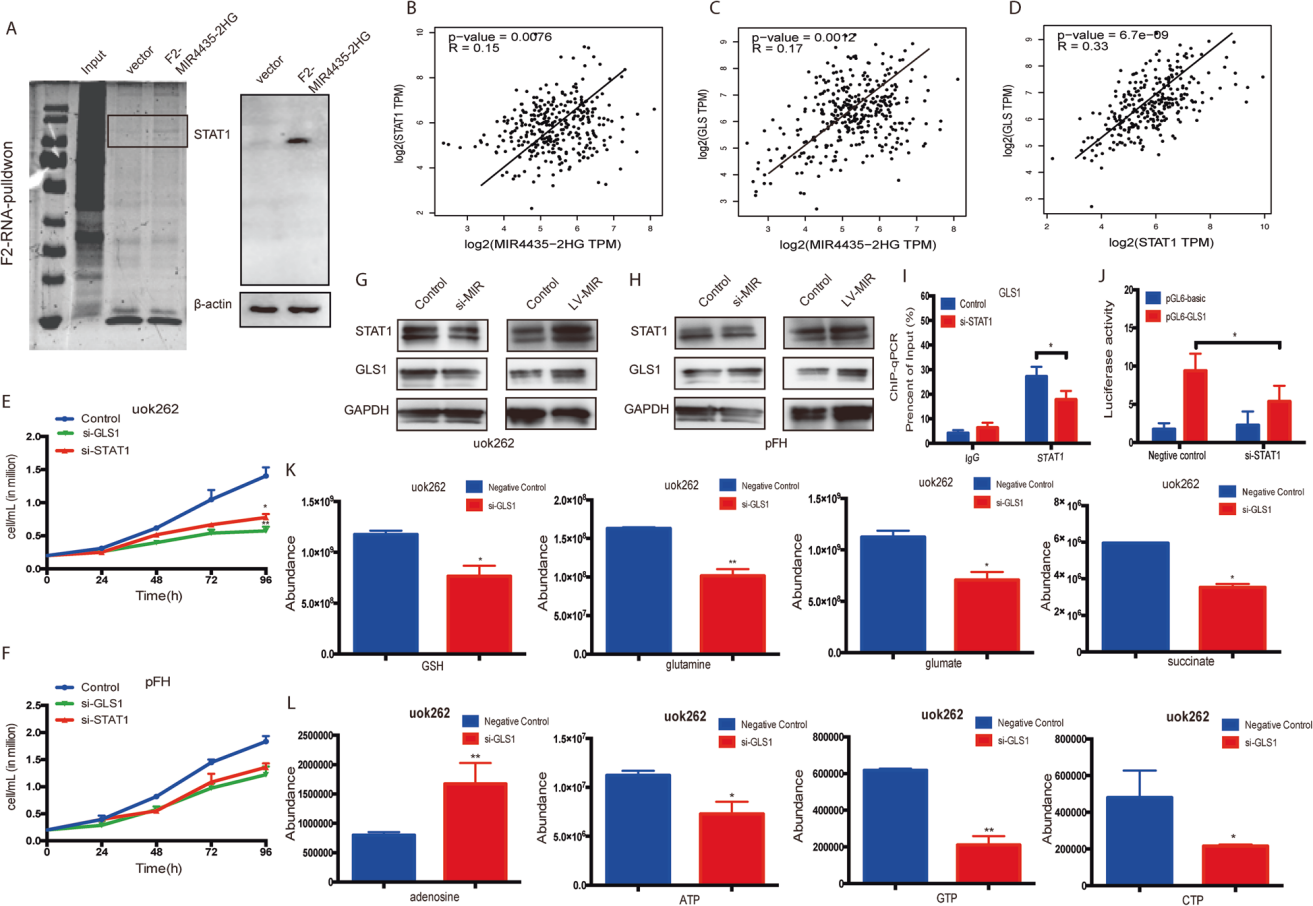
MIR4435-2HG could specifically bind to STAT1 and transcript activated GLS1. **A** RNA-pulldown assay was performed to determine the binding protein of MIR4435-2HG. **B**–**D** Public database was used to verify the positive correlation of MIR4435-2HG, STAT1 and GLS1 in pRCC. **E**, **F** Cell proliferation assay was performed in UOK262 and pFH cells that transfected with si-GLS1 and si-STAT1. **G**, **H** Western-blot assay was used to determine the correlation of STAT1 and GLS1 in cell lines. **I**, **J** ChIP and luciferase assays were performed to analyze the binding activation of STAT1 and GLS1. **K**, **L** TCA and nucleotide metabolites detection were performed by Mass spectrometry after GLS1 knocking down *p < 0.05, **p < 0.01, ***p < 0.001.

To determine the detailed mechanism of MIR4435-2HG in carcinogenesis, we used bioinformatics analysis to find the direct the signal pathway and RNA-binding proteins in the first place. The results showed that MIR4435-2HG could directly interact with several transcription factors, such as SAMD1, SAMD4, STAT1, MYC, SOX2 and so on (Supplementary Table S6). Then we applied the RNA-pulldown assay combined with western-blot experiments to screen the LncRNA-interacting proteins, and ChIRP experiment found that MIR4435-2HG could direct bind to STAT1 (Fig. 4A, Supplementary Fig. S3A). Previous study has reported that STAT1 would transcriptional activate the glutaminase 1 (GLS1), the rate-limiting enzyme of glutamine decomposition process. The relation of MIR4435-2HG and STAT1/GLS1 was further analyzed by GEPIA database, the results showed that MIR4435-2HG was positively correlated with STAT1 and GLS1 respectively, and STAT1 was significantly correlated with GLS1 either (Fig. 4B–D). To identify the importance of MIR4435-2HG/STAT1/GLS1 signal in FH-deficient RCC, we constructed the siRNA-STAT1 and siRNA-GLS1, and transfected with UOK262/pFH cells respectively. The results showed that both STAT1 and GLS1 knocking down could decrease the proliferation of UOK262 cells (Fig. 4E, F). Western-blot showed that STAT1 knocking down could decrease the GLS1 expression in UOK262 cells (Fig. 4G, H). ChIP-qPCR assay was also performed to verify the specific binding of STAT1 and GLS1. We found that STAT1 as a transcription factor can specifically target to the promoter of GLS1 (Fig. 4I, J, Supplementary S3B). Further, we tested the metabolites of TCA cycle and nucleotide metabolism, the MS results showed that GLS1 knocking down significantly suppressed the downstream TCA cycle process and inhibited ATP production (Fig. 4K, L, Supplementary Fig. S3C, D). Which means glutamine metabolism is critical in FH-deficient RCC’s proliferation. The above data showed that MIR4435-2HG could recruit STAT1 for transcription activate GLS1, thus promote the glutamine metabolism remodeling. GLS1 might be a promising therapeutic target in FH-deficient RCC patients.

### GLS1 specific inhibitor CB-839 suppressed the proliferation of FH-deficient RCC in vitro and in vivo

**Fig. 5:**
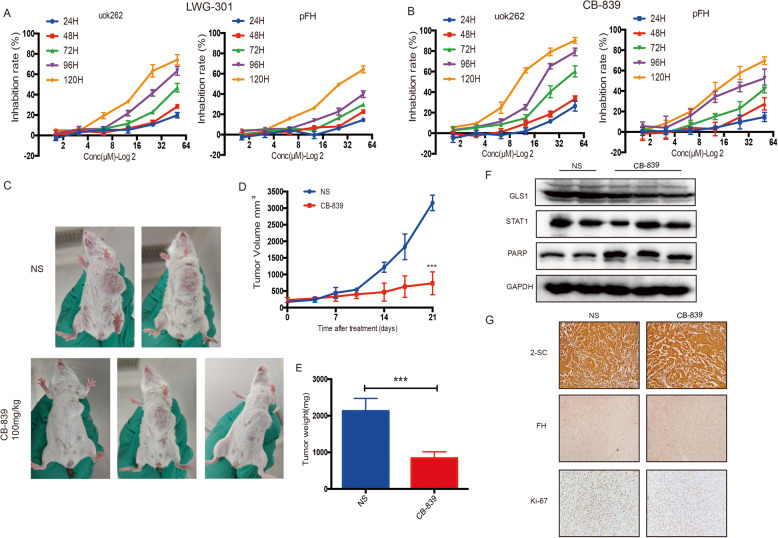
GLS1 inhibitor could significantly decrease the proliferation of FH-deficient RCC in vitro and in vivo. **A**, **B** The inhibition rate of GLS1 inhibitor LWG-301 and CB-839 in UOK262 and pFH cells. **C**, **D** Tumor volume was measured in CB-839 intragastric therapy or normal saline group. **E** Tumor weight was measured in the end of in vivo experiment. **F** Western-blot assay was performed to determine the GLS1, STAT1 and PARP expression in PDX-tumors. **G** The 2-SC, FH and Ki-67 expression detection based on PDX tissues IHC analysis. *p* < 0.05, **p < 0.01, ***p < 0.001.

**Fig. 6:**
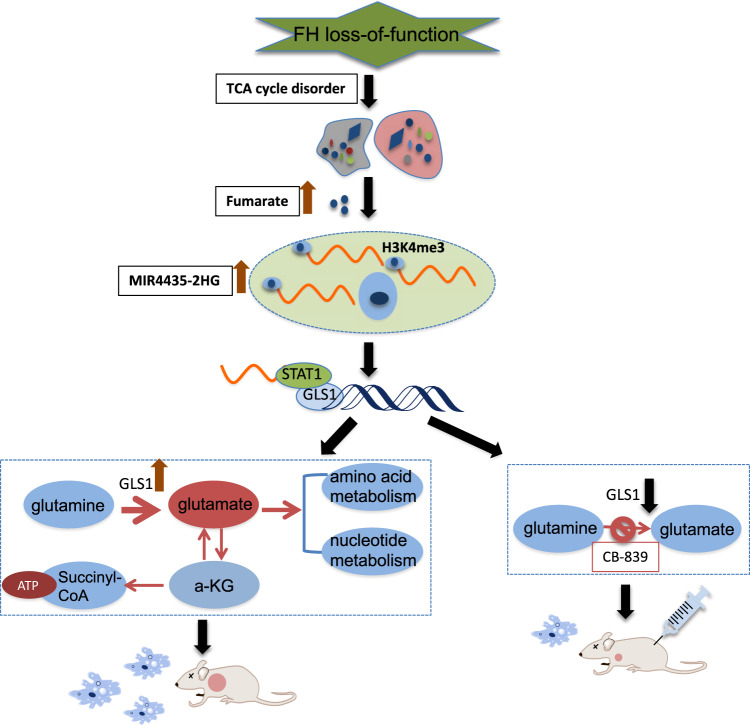
Schematic diagram of this research.

We sought to determine the therapeutic potential of GLS1 inhibitor for FH-deficient RCC. Firstly, we tested two GLS1 inhibitors (CB-839 and LWG-301, brought from MCE) in UOK262 and pFH cells independently. The anticancer effects were shown in Fig. 5A, B, compared with pFH cells UOK262 showed significant drug sensitivity to GLS1 inhibitors, and showed significant concentration and time dependence. CB-839 was shown better inhibitory effect than LWG-301, and selected for in vivo experiments. Patient-derived tumor xenograft (PDX) models of HLRCC were well established and 5 PDX mice were randomized divided into treatment group (3 mice) and control group (2 mice) (Patient’s information was collected in supplementary). Mice were treated with CB-839 (100 mg/kg) or normal saline orally twice a week for three weeks. We used tumor size and tumor weight as the surrogate for tumor burden, the tumor size were monitored and measured twice a week, the PDX tumors were harvested and weighted in the end of in vivo experiments. The results showed that the average tumor volume in CB-839-treated group was significantly smaller than control group (Fig. 5C–E), while the body weight was comparable means there was no severe toxicity reaction. The western-blot by using tumor protein showed that CB-839 treatment decreased GLS1 and elevated PARP expression (Fig. 5F). IHC staining confirmed two specific biomarkers 2-SC and FH in PDX-mouse models, and Ki-67 expression showed CB-839 inhibited cell proliferation (Fig. 5G). Thus, we conclude that GLS1 inhibitor CB-839 is effective in FH-deficient RCC. In summary, we provide a schematic diagram to help illustrate the detailed mechanism of fumrate induced MIR4435-2HG expression and MIR4435-2HG/STAT1/GLS1 signal pathway in FH-deficient RCC, and GLS1 inhibitor might serve as a promising therapeutic target (Fig. 6).

## Discussion

In recent years, the diagnosis and treatment of FH-deficient RCC has gradually become a hotspot and difficulty in the field of renal tumor. Several studies have reported new sights on this kind of tumor, both clinically and in scientific research, however, the potential involvement of LncRNA is poorly defined in FH-deficient RCC. The metabolomics has found that the crosstalk between LncRNA and tumor cell metabolism is closely related to the progression and metastasis of cancer. Exploring the regulatory mechanism of LncRNA and metabolism will provide a new perspective for drug development. Through literature study, we found that the regulatory network between LncRNA and metabolism was a key step in the occurrence and development of tumor. For example, LncRNA-p21 has been confirmed to be an important regulatory factor in the occurrence of Warburg effect in the process of cell carcinogenesis [18]. LncRNA-BCAR4 has been found to be an important target downstream of the YAP pathway in promoting the progression of glycolysis in breast cancer [19]. LncRNA-GLCC1 can promote the occurrence of colorectal cancer by stabilizing c-Myc regulating glucose metabolism [20]. In our study, through a combination of genomic, biochemical and cell biological analyses, we found an oncogenic LncRNA-MIR4435-2HG in FH-deficient RCC. We found that in cultured cell research MIR4435-2HG inhibition could significantly decrease the proliferation and inhibit glutamine metabolism progression in FH-deficient RCC cells. In terms of mechanism exploration, we found that fumarate could increase MIR4435-2HG expression by regulating H3K4me3 modification. Cut&tag sequencing was performed in this study as well, and we found that MMF incubating could active several pathways, such as MAPK signal pathway (Supplementary Fig. S4). While MIR4435-2HG was found to specifically bind STAT1, and then transcriptional activate GLS1 expression. Public database have also confirmed that MIR4435-2HG was positively correlated with the expression of STAT1 and GLS1. In xenograft mouse models, GLS1 inhibitor CB-839 could markedly suppress tumor growth. These data consistently revealed that MIR4435-2HG participated in the regulation of glutamine metabolism, and promoted the energy remodeling and malignant development of FH-deficient RCC, and GLS1 could be metabolically target for these tumors.

Tumor metabolism has been shown to play a central role in the control and regulation of cancer cell plasticity. Following disruption of metabolic pathways or activation of oncogenic pathways, cancer cells can undergo metabolic reprogramming to adapt their metabolism to the energy and anabolic requirements necessary for uncontrolled proliferation and migration [21]. It is well known that FH loss impairs TCA cycle activity and induces fumarate accumulation in FH-deficient RCC. The fumarate was proved to be a kind of truly carcinogenic metabolite, and has been shown to have many cancer-promoting functions, including inhibited the antimetastasic miRNA cluster by Tet-mediate demethylation and leaded to EMT-related factors’ activation [21], caused genes hypermethylation by inhibiting histone and DNA demethylases [12], induced urea cycle remodeling [22], and so on. In our study, we used HLRCC tissue samples for metabolic mass spectrometry detection, and found that glutamine metabolism was obviously active in HLRCC. Combined with previous in vitro and in vivo data, we suggest that fumarate activates glutamine metabolism-related enzymes by inhibiting histone demethylase activity, thus leading to glutamine metabolic remodeling. GLS1 acts as the rate-limiting enzyme in the process of glutamine decomposition, and hydrolyzes glutamine into glutamate and ammonia after glutamine enters the cell, while glutamate can be further transformed into a-ketoglutarate into the TCA cycle or directly into the amino acid cycle and nucleotide synthesis as a nitrogen source [23]. This process also provides energy for cell proliferation. Studies have shown that GLS1 has an important relationship with tumor proliferation and angiogenesis [24, 25]. Currently, several GLS1 inhibitors have been used in clinical trials related to tumor therapy and have shown certain tumor inhibition effect, including lymphoma [26], pancreatic cancer [27] and breast cancer [28]. However, studies on glutamine metabolism and related inhibitors have not been well reported in FH-deficient RCC. Based on previous data, we believes that remodeling of glutamine metabolism also plays an important role in the development of FH-deficient RCC, and the application of GLS1 inhibitors (such as CB-839) may benefit these patients. What’s more, MIR4435-2HG will be an important drug sensitivity marker for GLS1 targeted therapy.

### Supplementary information

SUPPLEMENTARY

Original Data File

Supplementary Figures and tables legends

checklist

## Data Availability

All data are available upon reasonable request.
