# Supplementary material for: Fumarate induces LncRNA-MIR4435-2HG to regulate glutamine metabolism remodeling and promote the development of FH-deficient renal cell carcinoma
Source: Cell Death Dis. 2024 Feb 19;15(2):151. doi: 10.1038/s41419-024-06510-2 (PMC10876950; doi:10.1038/s41419-024-06510-2)
Supplement: Supplementary file 1 — SUPPLEMENTARY [file 41419_2024_6510_MOESM1_ESM.pdf]

Supplementary

Figure S1

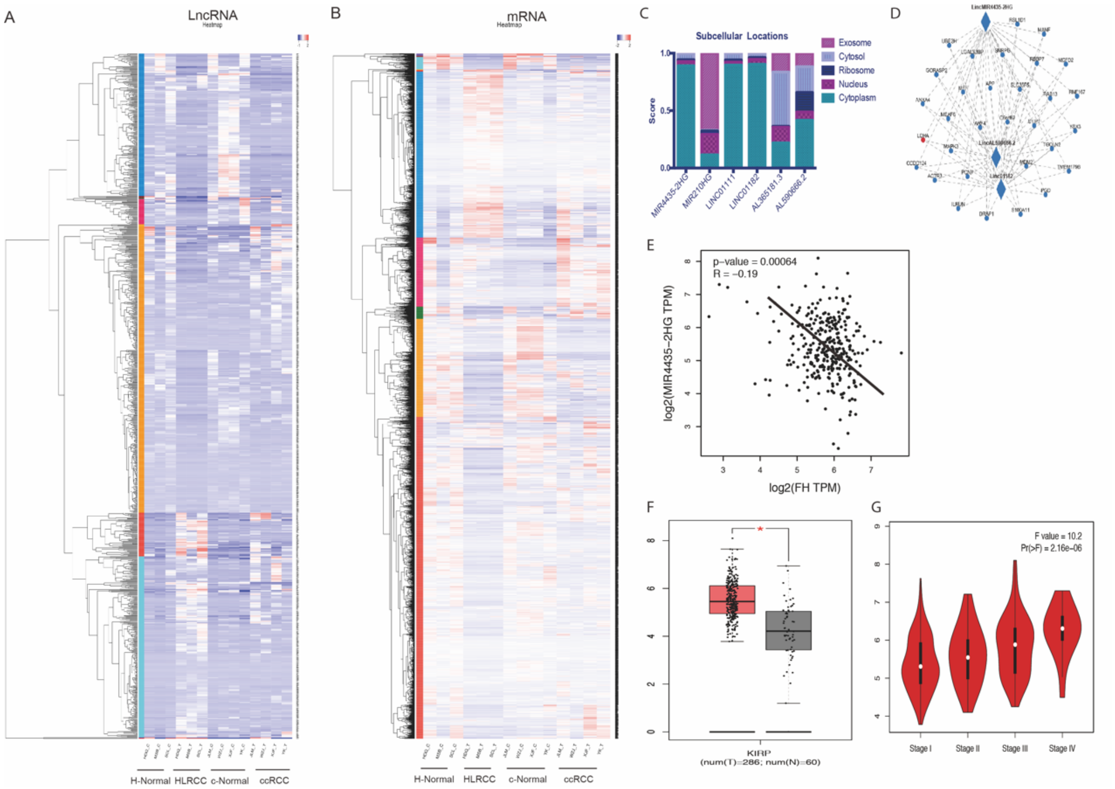

Figure S2

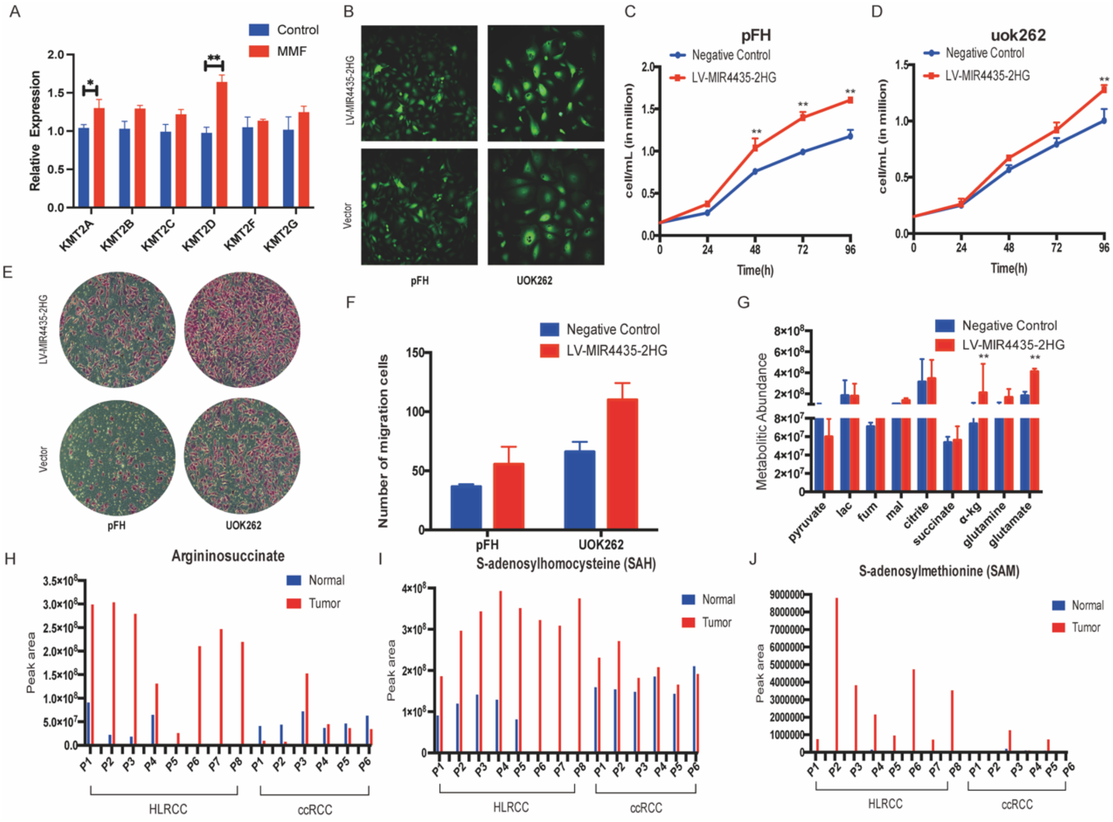
A
Control
MMF
Relative Expression
KMT2A
KMT2B
KMT2C
KMT2D
KMT2F
KMT2G
B
LV-MIR4435-2HG
Vector
pFH
UOK262
C
pFH
Negative Control
LV-MIR4435-2HG
cell/mL (in million)
Time(h)
D
uok262
Negative Control
LV-MIR4435-2HG
cell/mL (in million)
Time(h)
E
LV-MIR4435-2HG
Vector
pFH
UOK262
F
Negative Control
LV-MIR4435-2HG
Number of migration cells
pFH
UOK262
G
Negative Control
LV-MIR4435-2HG
Metabolitic Abundance
pyruvate
lac
fum
mal
citrite
succinate
α-kg
glutamine
glutamate
H
Argininosuccinate
Normal
Tumor
Peak area
HLRCC
ccRCC
I
S-adenosylhomocysteine (SAH)
Normal
Tumor
Peak area
HLRCC
ccRCC
J
S-adenosylmethionine (SAM)
Normal
Tumor
Peak area
HLRCC
ccRCC

Figure S3

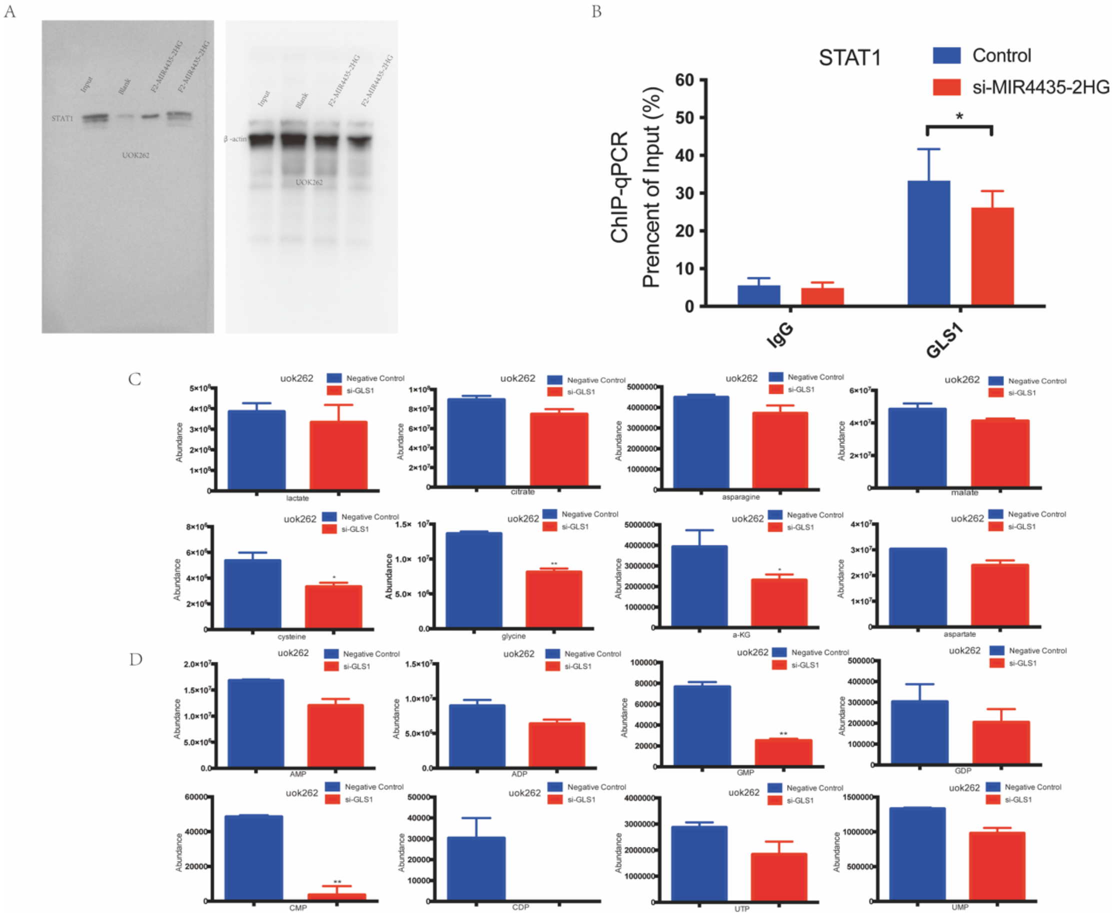
A
Input
Blank
F2-MIR4435-2HG
F2-MIR4435-2HG
STAT1
UOK262
β-actin
UOK262
B
STAT1
Control
si-MIR4435-2HG
ChIP-qPCR
Prencent of Input (%)
60
50
40
30
20
10
0
*
IgG
GLS1
C
uok262
Negative Control
si-GLS1
Abundance
lactate
citrate
asparagine
malate
cysteine
glycine
a-KG
aspartate
D
AMP
ADP
GMP
GDP
CMP
CDP
UTP
UMP

Figure S4

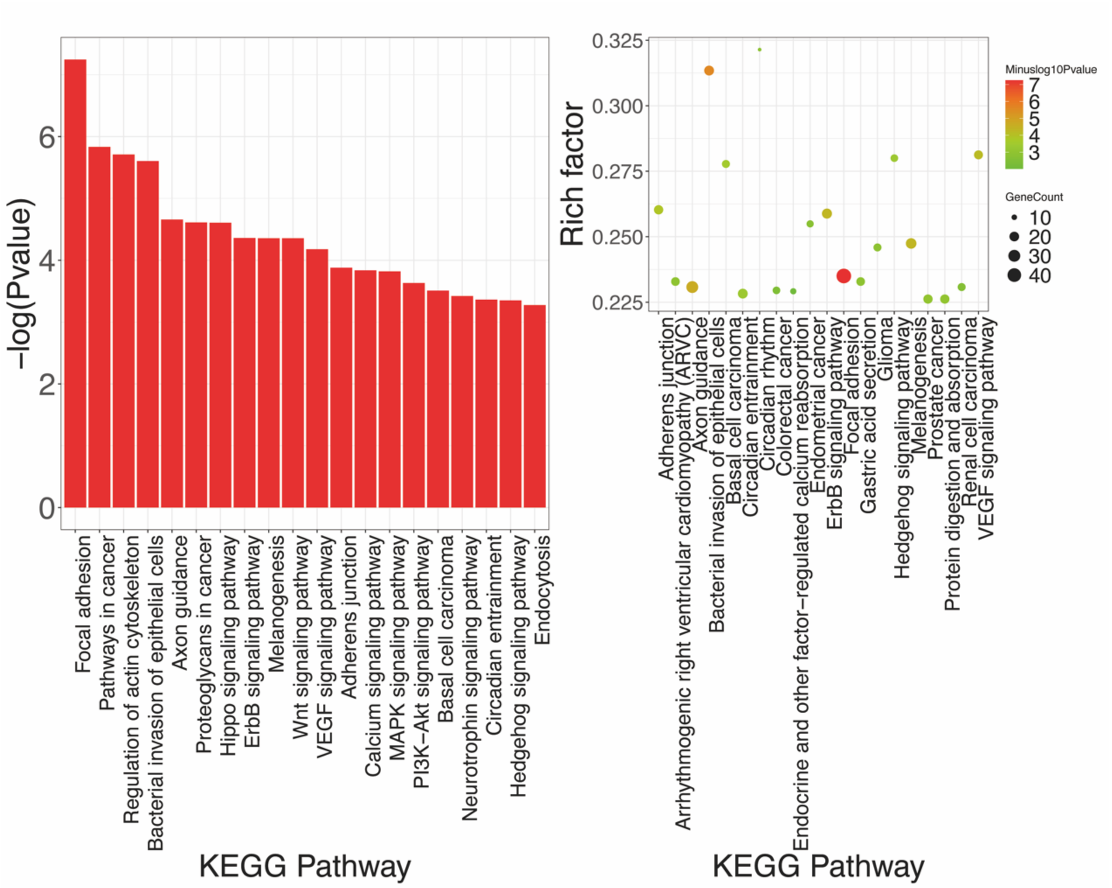

−log(Pvalue)
KEGG Pathway
Focal adhesion
Pathways in cancer
Regulation of actin cytoskeleton
Bacterial invasion of epithelial cells
Axon guidance
Proteoglycans in cancer
Hippo signaling pathway
ErbB signaling pathway
Melanogenesis
Wnt signaling pathway
VEGF signaling pathway
Adherens junction
Calcium signaling pathway
MAPK signaling pathway
PI3K−Akt signaling pathway
Basal cell carcinoma
Neurotrophin signaling pathway
Circadian entrainment
Hedgehog signaling pathway
Endocytosis
Rich factor
Minuslog10Pvalue
GeneCount
Adherens junction
Arrhythmogenic right ventricular cardiomyopathy (ARVC)
Axon guidance
Bacterial invasion of epithelial cells
Basal cell carcinoma
Circadian entrainment
Circadian rhythm
Colorectal cancer
Endocrine and other factor−regulated calcium reabsorption
Endometrial cancer
ErbB signaling pathway
Focal adhesion
Gastric acid secretion
Glioma
Hedgehog signaling pathway
Melanogenesis
Prostate cancer
Protein digestion and absorption
Renal cell carcinoma
VEGF signaling pathway
KEGG Pathway

Supplementary Table

Table S1

N	Gender	pathology	TNM
1	female	type II pRCC, grade III	T3aN1M0
2	male	type II pRCC, grade II-III	T1bN0M0
3	male	type II pRCC, grade III	T1bN1M0
4	female	type II pRCC, grade II-III	T3bN0M0
5	female	type II pRCC, grade II	T1bN0M0
6	female	type II pRCC, grade III	T3bN1M0
7	male	type II pRCC, grade II	T2aN0M0
8	male	type II pRCC, grade III	T1bN1M0
9	male	ccRCC, grade II	T1bN0M0
10	female	ccRCC, grade II	T1aN0M0
11	male	ccRCC, grade I	T2aN0M0
12	male	ccRCC, grade III	T2aN0M0
13	male	ccRCC, grade I	T2bN1M0
14	female	ccRCC, grade II	T2bN0M0

Table S2

GENE	FISH Probe sequence
MIR4435-2HG-1	AGGCATTAAGTCAAGTCCAGGTTTGTCAGTTTCTCCA
MIR4435-2HG-2	GCAGCGACCATCCAGTCATTTATTTCCCTCCATTC

Table S3

LncRNA	5'-3'		HMTs	5'-3'
MIR4435-2HG			KMT2A	
F	AGAAGCCACTGGAGCAGAAGG		F	GCAGCGGAGAGGATGAGCAATTC
R	AGACAAGGTTCACAAGTTGGTAAGG		R	TTCGGTCAGAGCCACTTCTAGGTC
MIR210HG			KMT2B	
F	CATATCTTCAGCCAACAGGACCATC		F	CCTTCCTCCTCCTCGCCTAGC
R	CAGAAACACACAGAAGCACCAAATC		R	CACCATCCGTTCTGTGCCTTCC
LINC01111			KMT2C	
F	GAAGAGCAAGTAGGATGGTGAAGTG		F	CGTTTACAGCCAGGTGAGGAAGTG
R	TGAGTGAGCATCGGCAGTAGC		R	AGTGGACTCCTGACCGTTGACATC
LINC01182			KMT2D	
F	CGATGCCTTTCCTGGTGGTTTC		F	TGCCAGCGGTTCCTTCCTATCC
R	CATTGTAAGGAAGGACTTGGCTGAG		R	ACAGCGAGCCTCCTCCAGATATG
AL590666.2			KMT2F	
F	GTATTCCAGCTTCAGCCTGTCTAC		F	TCCCGCCGCCACTTCTCTG
R	TCAGGTATCTATCGCTGTGAGGTC		R	ACGAGGAGGACGAGGACAATGAG
AL365181.3			KMT2G	
F	TGAAGGTTAGACTACAGGCAGGAC		F	AGCCCACACCCTCATACCTCTTC
R	AGGGACAGGTATGAAGGCAGATG		R	GCGTCCGTGAACTTGCTCTCG

Table S4

siRNA	5'-3'
LINC02532 F	GGAUGUGAGUGUGUAGACCUUTT
LINC02532 R	AAGGUCUACACACUCACAUCCTT
MIR210HG F	GGAAGAGAGUGACAGAUUUTT
MIR210HG R	AAAUCUGUCACUCUCUUCCCG
LINC01111 F	GGUGCAUCAAAGAAGGUAATT
LINC01111 R	UUACCUUCUUUGAUGCACCAT
LINC01559 F	GGGAAAUCCACUAAGACUAGATT
LINC01559 R	UCUAGUCUUAGUGGAUUUCCCTT
LINC01182 F	GCCUCCAGACUCUUAUACUGUTT
LINC01182 R	ACAGUAUAAGAGUCUGGAGGCTT
MIR4435-2HG F	GGAUGGUGACUUGGACUUGUATT
MIR4435-2HG R	UACAAGUCCAAGUCACCAUCCTT
STAT1-1F	UGGAUUUGUACCAUUCUUCUG
STAT1-1R	GAAGAAUGGUACAAAUCCAAG
STAT1-2F	ACUCAUUGGUUCCUUUAAGGG
STAT1-2R	CUUAAAGGAACCAAUGAGUCC
GLS1-1F	AUAGAACAGCAAAUCUUCCAA
GLS1-1R	GGAAGAUUUGCUGUUCUAUAC
GLS1-2F	UAUAGAACAGCAAAUCUUCCA
GLS1-2R	GAAGAUUUGCUGUUCUAUACA

Table S5

```
>ENST00000409569.2 MIR4435-2HG-203 cdna:lncRNA     530bp    50%GC
GAAGGCTGAGGTGTGCGCCTTTTTTTTTTTTCCTTCTTAGTCGTGTGTACATCATTGGGAATGGAG
GGAAATAAATGACTGGATGGTCGCTGCTTTTTAAGTTTCAAATTGACATTCCAGACAAGCGGTG
CCTGAGCCCGTGCCTGTCTTCAGATCTTCACAGCACAGTTCCTGGGAAGGTGGAGCCACCAGCC
TCTCCCTGAATAACTGGGAGATGAAACAGGAAGCTCTATGACACACTTGATCGAATATGACAGA
CACTGAAAATCACGACTCATCCCCCTCCAGCACCTCTACCTGTTGCCCGCCGATCACAGCCGGA
ATGCAGCTGAAAGATTCCCTGGGGCCTGGTTCCAACTGCCCACTGTGGACTCTGAGGCCTCTGC
ATTTGCGGGTGGTCTGCCTGTGATATTTTGGTCATGGGCTGGTCTGGTCGGTTTCCCATTTGTCTG
GCCAGTCTCTGTGTGTCTTAATCCCTTGTCCTTCATTAAAAGCAAAACTAAAGAAAACAGAAGGC
GCTGACAAAGCGC
```

批注 [A1]: F2-RNA

Table S6

gene	lncRNA	RNA_Binding_Protein	RNAInter	IF
NANOG	MIR4435-2HG	RNA_Binding_Protein	RNAInter	1
OTX2	MIR4435-2HG	RNA_Binding_Protein	RNAInter	1
POLR2A	MIR4435-2HG	RNA_Binding_Protein	RNAInter	0
SMAD1	MIR4435-2HG	RNA_Binding_Protein	RNAInter	1
SMAD4	MIR4435-2HG	RNA_Binding_Protein	RNAInter	1
SNAI2	MIR4435-2HG	RNA_Binding_Protein	RNAInter	1
SRF	MIR4435-2HG	RNA_Binding_Protein	RNAInter	1
STAT1	MIR4435-2HG	RNA_Binding_Protein	RNAInter	1
TAL1	MIR4435-2HG	RNA_Binding_Protein	RNAInter	1
MYC	MIR4435-2HG	RNA_Binding_Protein	RNAInter	1
CHD8	MIR4435-2HG	RNA_Binding_Protein	RNAInter	0
CHD7	MIR4435-2HG	RNA_Binding_Protein	RNAInter	0
SOX2	MIR4435-2HG	RNA_Binding_Protein	RNAInter	1
POU5F1	MIR4435-2HG	RNA_Binding_Protein	RNAInter	1
MRE11	MIR4435-2HG	RNA_Binding_Protein	RNAInter	0
UBTF	MIR4435-2HG	RNA_Binding_Protein	RNAInter	1
CASP1	MIR4435-2HG	RNA_Binding_Protein	RNAInter	0
CTCF	MIR4435-2HG	RNA_Binding_Protein	RNAInter	1
STAT3	MIR4435-2HG	RNA_Binding_Protein	RNAInter	1
