# Supplementary figures and images for: Fumarate induces LncRNA-MIR4435-2HG to regulate glutamine metabolism remodeling and promote the development of FH-deficient renal cell carcinoma
Source: Cell Death Dis. 2024 Feb 19;15(2):151. doi: 10.1038/s41419-024-06510-2 (PMC10876950; doi:10.1038/s41419-024-06510-2)

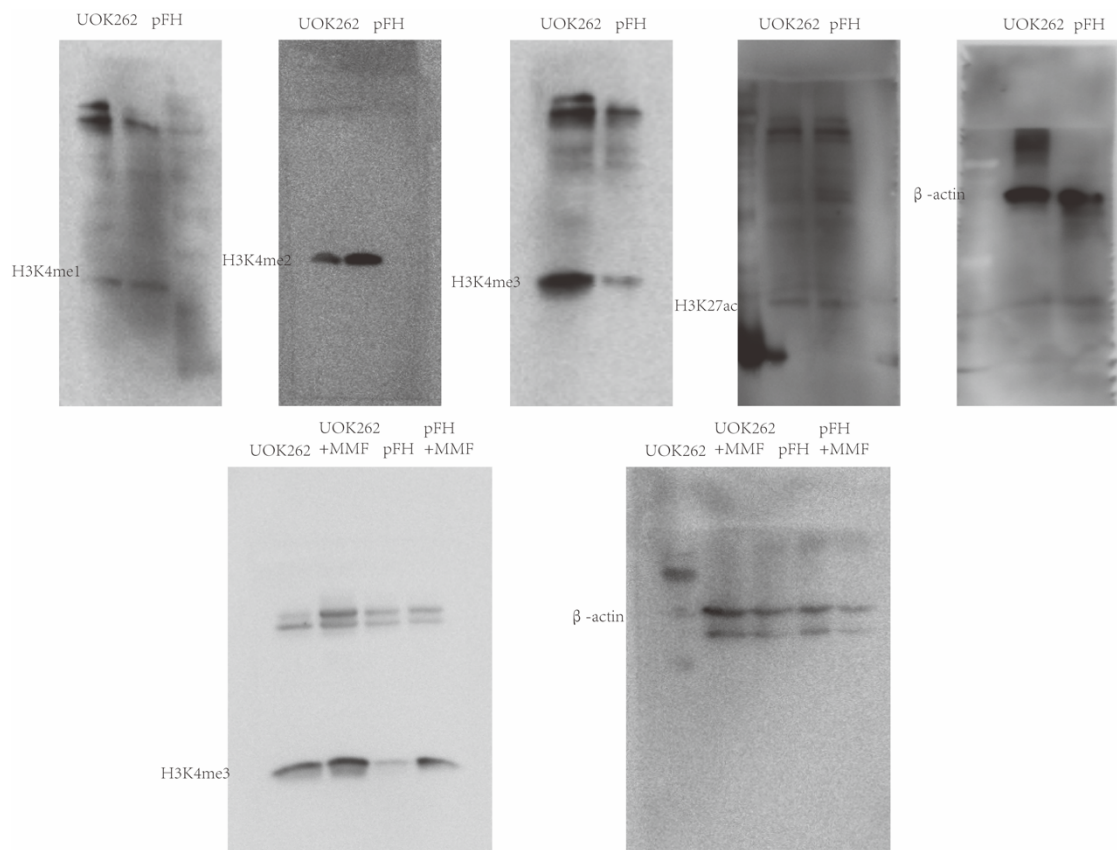

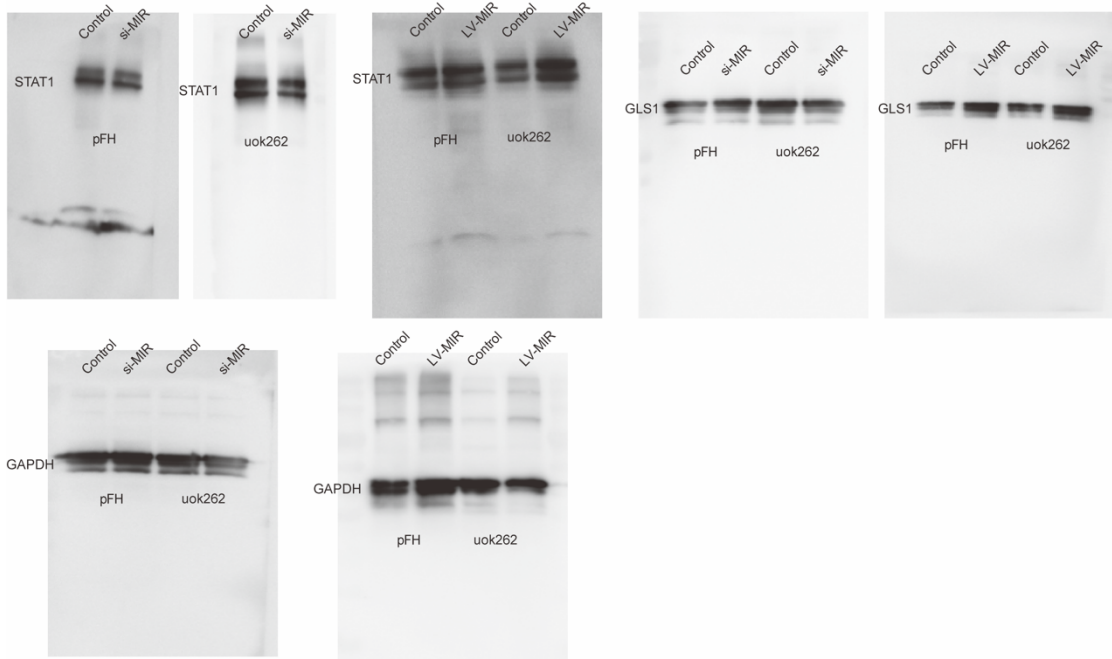

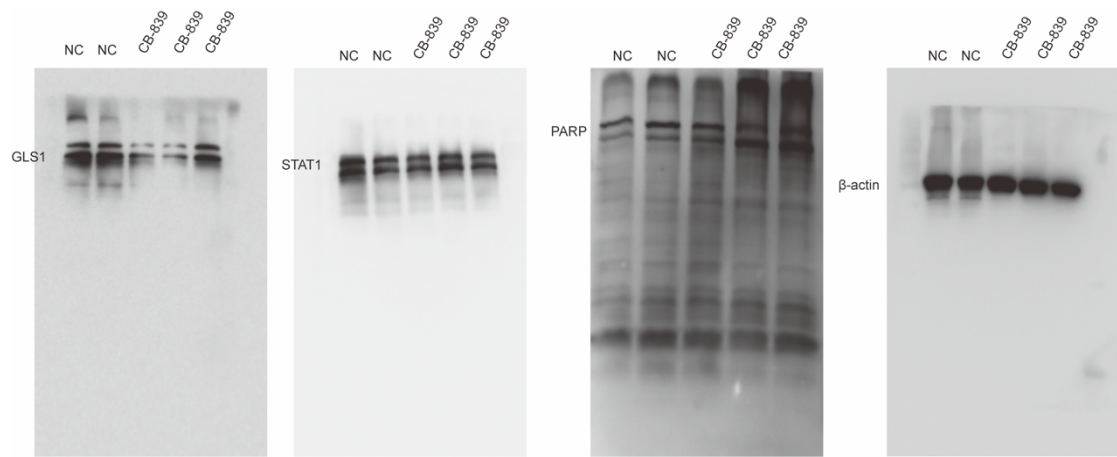

Supplement: Supplementary file 2 — Original Data File [file 41419_2024_6510_MOESM2_ESM.pdf]
