# Supplementary material for: Fumarate induces LncRNA-MIR4435-2HG to regulate glutamine metabolism remodeling and promote the development of FH-deficient renal cell carcinoma
Source: Cell Death Dis. 2024 Feb 19;15(2):151. doi: 10.1038/s41419-024-06510-2 (PMC10876950; doi:10.1038/s41419-024-06510-2)
Supplement: Supplementary file 3 — Supplementary Figures and tables legends [file 41419_2024_6510_MOESM3_ESM.docx]

**Supplementary Figure legends**

Figure S1. The bioinformatics analysis result. A-B The LncRNA and mRNA profile of FH-deficient RCC sequencing. C The sublocation of selected LncRNAs. D The correlation of LncRNAs and mRNAs. E The correlation of FH and MIR4435-2HG according to the GEPIA database. F-G The expression level of MIR4435-2HG in KIRP data.

Figure S2. A The expression of H3K4 methyl transferase after MMF treatment. B The representative images of lentivirus transfection. C-D The proliferation of LV-MIR4435-2HG and negative control in Pfh and UOK262 cells. E-F The transwell assay showed MIR4435-2HG improved cell migration. G The MS analysis showed specific metabolite after MIR4435-2HG overexpression. H-J The argininesuccinate, SAH and SAM level in HLRCC and ccRCC samples. p<0.05, **p<0.01, ***p<0.001.

Figure S3. A The ChIRP experiment showed MIR4435-2HG could specifically bind to STAT1. B The ChIP-qpcr assay showed MIR4435-2HG knocking down could decrease the binding activity of STAT1 and GLS1 promoter. C-D The MS analysis tested the metabolites of TCA cycle (A) and nucleotide metabolism (B). p<0.05, **p<0.01, ***p<0.001.

Figure S4. The representative ChIP-sequencing data of FH-deficient cancer cells after MMF incubating.

**Supplementary Table legends**

Tbale S1. The patients’ information of cancer samples that used in this study.

Table S2. The FISH probe sequence of MIR4435-2HG.

Table S3. The qPCR sequence of LncRNAs and HMTs in this study.

Table S4. The siRNA sequence of LncRNAs, STAT1 and GLS1.

Table S5. The structure and sequence of F2-MIR4435-2HG probe.

Table S6. The TOPs RNA-binding proteins of MIR4435-2HG.
